# A CT-based radiomics nomogram for differentiation of focal nodular hyperplasia from hepatocellular carcinoma in the non-cirrhotic liver

**DOI:** 10.1186/s40644-020-00297-z

**Published:** 2020-02-24

**Authors:** Pei Nie, Guangjie Yang, Jian Guo, Jingjing Chen, Xiaoli Li, Qinglian Ji, Jie Wu, Jingjing Cui, Wenjian Xu

**Affiliations:** 1 Department of Radiology, the Affiliated Hospital of Qingdao University, No.16, Jiangsu Road, Qingdao, 266000 Shandong China; 2 Department of Nuclear Medicine, the Affiliated Hospital of Qingdao University, Qingdao, Shandong China; 3 Department of Pathology, the Affiliated Hospital of Qingdao University, Qingdao, Shandong China; 4 Huiying Medical Technology Co., Ltd, Beijing, China

**Keywords:** Focal nodular hyperplasia, Hepatocellular carcinoma, Tomography, X-ray computed, Radiomics

## Abstract

**Background:**

The purpose of this study was to develop and validate a radiomics nomogram for preoperative differentiating focal nodular hyperplasia (FNH) from hepatocellular carcinoma (HCC) in the non-cirrhotic liver.

**Methods:**

A total of 156 patients with FNH (*n* = 55) and HCC (*n* = 101) were divided into a training set (*n* = 119) and a validation set (*n* = 37). Radiomics features were extracted from triphasic contrast CT images. A radiomics signature was constructed with the least absolute shrinkage and selection operator algorithm, and a radiomics score (Rad-score) was calculated. Clinical data and CT findings were assessed to build a clinical factors model. Combined with the Rad-score and independent clinical factors, a radiomics nomogram was constructed by multivariate logistic regression analysis. Nomogram performance was assessed with respect to discrimination and clinical usefulness.

**Results:**

Four thousand two hundred twenty-seven features were extracted and reduced to 10 features as the most important discriminators to build the radiomics signature. The radiomics signature showed good discrimination in the training set (AUC [area under the curve], 0.964; 95% confidence interval [CI], 0.934–0.995) and the validation set (AUC, 0.865; 95% CI, 0.725–1.000). Age, Hepatitis B virus infection, and enhancement pattern were the independent clinical factors. The radiomics nomogram, which incorporated the Rad-score and clinical factors, showed good discrimination in the training set (AUC, 0.979; 95% CI, 0.959–0.998) and the validation set (AUC, 0.917; 95% CI, 0.800–1.000), and showed better discrimination capability (*P* < 0.001) compared with the clinical factors model (AUC, 0.799; 95% CI, 0.719–0.879) in the training set. Decision curve analysis showed the nomogram outperformed the clinical factors model in terms of clinical usefulness.

**Conclusions:**

The CT-based radiomics nomogram, a noninvasive preoperative prediction tool that incorporates the Rad-score and clinical factors, shows favorable predictive efficacy for differentiating FNH from HCC in the non-cirrhotic liver, which might facilitate clinical decision-making process.

## Background

Hepatocellular carcinoma (HCC) is the most common primary liver cancer and the third most common cause of cancer death worldwide [1, 2]. Approximately 80% of cases of HCC occur in patients with liver cirrhosis, arising from hepatitis B and C infections or alcoholism [2, 3]. In patients with liver cirrhosis, noninvasive diagnosis of HCC can be established by a characteristic feature of arterial phase hyperenhancement followed by portal venous or delayed phase washout on multiphasic contrast CT or MRI. However, an increasing number of HCC arises in a non-cirrhotic liver [3], probably due to transient hepatitis B infection or due to diffuse liver damage caused by non-alcoholic fatty liver disease. In such non-cirrhotic cases, other benign hypervascular liver lesions (hepatocellular adenoma [HCA] and focal nodular hyperplasia [FNH]) should be taken into the differential diagnosis.

FNH is the second most common benign liver tumour in the non-cirrhotic liver, characterized by nodular hyperplasia of the hepatic parenchyma around a central stellate area of fibrosis associated with a congenital vascular malformation [4–7]. Typical FNH can be diagnosed with confidence by using multiphasic contrast CT or MRI. Atypical FNH may show less intense enhancement, absence of a central scar, pseudocapsular enhancement on delayed images, as well as the presence of hemorrhage, calcification, or necrosis [8, 9], making the differential diagnosis between atypical FNH and HCC rather difficult. The distinction between HCC and FNH is critical as the management differs considerably.

Various imaging modalities have been applied in the distinction between HCC and FNH, such as CT [1, 9, 10], Doppler ultrasound [11, 12] and MRI [1, 3, 5, 13–15]. In previous studies, the gadoxetic acid-enhanced MRI is being increasingly used in differentiating focal liver lesions. HCC generally shows definite hypointensity on the hepatobiliary phase (HBP) because of decreased or absent uptake of gadoxetic acid. On the other hand, FNH commonly shows iso- or hyperintensity on the HBP because of their preserved ability to take up gadoxetic acid. However, 10–15% of HCCs show iso- or hyperintensity on the HBP because of overexpression of organic anion-transporting polypeptide (OATP) 1B3, which is one of the uptake transporters of gadoxetic acid into hepatocytes [1]. Approximately 10–12% of FNHs may not show iso- or hyperintensity on the HBP [7]. The paradoxical uptake or lack of uptake may make the differential diagnosis of HCC from FNH rather difficult.

Radiomics, as an emerging field involved with the extraction of high-throughput data from quantitative imaging features and the subsequent combination of this information with clinical data, has the potential to provide diagnostic, prognostic, and predictive information and improve clinical decision making [16, 17]. Successful applications of radiomics in liver tumours have been reported in prediction of histologic grade, recurrence, liver failure and survival after curative treatment or chemotherapy in HCC patients [18–33], in preoperative prediction of HCC microvascular invasion [34–36], in differentiating benign hepatic lesions (including hepatic haemangioma [HH], HCA, FNH, and hepatic abscess) from malignant tumours (including HCC and metastases) [37–41] and in discriminating different benign (HCA and FNH) [42, 43] or malignant liver tumours (HCC, intrahepatic cholangiocarcinoma [ICC] and combined HCC-ICC) [44]. To the best of our knowledge, few studies focused on radiomics in differentiating HCC from FNH in non-cirrhotic patients.

The purpose of this study was to construct and validate a CT based radiomics nomogram that would incorporate a radiomics signature and clinical factors for the preoperative differentiation between HCC and FNH in the non-cirrhotic liver.

## Methods

### Patients

The institutional review board of our hospital approved this retrospective study with a waiver of obtaining informed consent.

Patients were identified by searching the pathology database from one institution (The Affiliated Hospital of Qingdao University) between June 2008 and February 2019 for the diagnosis of FNH or HCC on surgically resected specimens. A total of 156 patients with FNH (*n* = 55, 32 men and 23 women; mean age, 31.82 ± 12.55 years) and HCC (*n* = 101, 85 men and 16 women; mean age, 57.10 ± 9.89 years) were enrolled in this study according to the following inclusion criteria: (1) patients with pathologically proven of either FNH or HCC; (2) patients underwent contrast-enhanced CT less than 15 days before surgery; (3) patients with complete clinical and pathologic data. The exclusion criteria were as follows: (1) HCC patients with CT features of liver cirrhosis (The cirrhotic liver may demonstrate a nodular surface, widened fissures between lobes, an atrophied right lobe, hypertrophy of left lobe and/or caudate lobe and other features including portal vein dilation, portosystemic shunts, splenomegaly, and ascites, etc.); (2) HCC patients received chemotherapy or radiotherapy before surgery; (3) Image quality was unsatisfactory for analysis. The patients were divided into two independent sets: 119 patients treated between June 2008 and January 2017 constituted the training set, whereas 37 patients treated between February 2017 and February 2019 constituted the validation set.

Clinical information including age, gender, hepatitis B and C virus (HBV and HCV) infection and serum alpha fetoprotein (AFP) level (> 400 ng/mL; ≤ 400 ng/mL) were derived from medical records.

### CT image acquisition

CT scans were obtained with two 64-slice CT scanners (Somatom Sensation 64, Siemens Healthcare, Erlangen, Germany; Discovery 750, GE Healthcare, Milwaukee, USA) using the following parameters: 120 kVp tube voltage, 200 mAs or 250–400 mA (using automatic tube current modulation) tube current, 64 × 0.6 mm or 64 × 0.625 mm detector collimation, a matrix of 512 × 512, a pitch of 1 or 1.375, a gantry rotation time of 0.5 s and a slice thickness of 5 mm. The scanning area covered the entire liver. An 80–90 mL volume of nonionic contrast agent (Iopromide, Ultravist 370; Bayer, Germany) was administered into the antecubital vein by a power injector at a rate of 2.5 mL/s. Pre-contrast CT was first obtained, followed by three post-contrast CT scans of the liver obtained in arterial phase (AP, 30 s), portal venous phase (PVP, 60 s), and delayed phase (DP, 90–120 s).

### CT features analysis

The CT images were analyzed in our Picture Archiving and Communication System (PACS, Version 3.2.8, GE Healthcare, Milwaukee, USA) by two radiologists (Reader 1, P.N; Reader 2, G.Y) with eight and 6 years of abdominal imaging experience, respectively. Blinded to the clinic-pathologic data, the two readers interpreted the following subjective CT features by consensus: the diameter of the tumour on the axial CT image; shape (round or not round); a central scar (present or absent, a “central scar” was defined as a central stellate structure showing low attenuation on unenhanced CT images, hypovascular enhancement on AP and PVP phases and delayed enhancement on DP phase); degeneration (present or absent, “degeneration” was considered as a non-enhancing area on dynamic study due to necrosis or hemorrhage. We supposed that low attenuation on unenhanced CT images corresponded to necrosis, whereas high attenuation on unenhanced CT images indicated hemorrhage); fat deposition (present or absent, “fat deposition” was defined as an area showing fat attenuation on unenhanced CT images); calcification (present or absent); a capsule-like rim (present or absent, “a capsule-like rim” was defined as tumour rim showing low attenuation on unenhanced CT images and hypovascular-delayed enhancement on dynamic studies); dysmorphic vessels (present or absent, “dysmorphic vessels” were regarded as prominent or enlarged vessels in or around the tumour); and enhancement pattern (The enhancement pattern on dynamic CT was classified into early enhancement with a washout pattern, early enhancement with no washout pattern and other patterns. Early enhancement was defined as showing higher attenuation than the background liver on AP. Washout was defined as a nodule showing lower attenuation than the background liver on PVP to DP. No washout pattern indicated that the nodule showed equivalent or higher attenuation than the background liver on PVP to DP. Other patterns referred to the enhancement patterns not mentioned above).

### Construction of the clinical factors model

Univariate analysis was applied to compare the differences of the clinical factors (including clinical information and CT features) between the two groups, and a multiple logistic regression analysis was used to build the clinical factors model using the significant variables from the univariate analysis as inputs. Odds ratios (OR) as estimates of relative risk with 95% confidence intervals (CI) were obtained for each risk factor.

### Tumour segmentation and radiomics feature extraction

Tumor regions of interest (ROIs) were manually segmented in the largest cross-sectional area using ITK-SNAP software (Version 3.8.0). Contouring was drawn slightly within the borders of the tumours on AP, PVP, and DP, but avoiding covering the adjacent hepatic parenchyma and perinephric fat.

Feature extraction was performed using the Radcloud platform (Huiying Medical Technology Co., Ltd). A total of 4227 radiomics features were extracted from the ROIs. The radiomics features are divided into four groups: (1) intensity statistics features, which consists of 19 features that quantitatively delineate the distribution of voxel intensities within the ROI through commonly used and basic metrics; (2) shape features, including 10 two-dimensional features, are used to reflect the shape and size of the ROI; (3) texture features, composed 59 features calculated by gray level co-occurrence matrix (GLCM), gray level run length matrix (GLRLM) and gray level size zone matrix (GLSZM), quantify the heterogeneity differences of ROI; and (4) filter and wavelet features, which include the intensity and texture features derived from filter transformation and wavelet transformation of the original image, obtained by applying filters such exponential, logarithm, square, square root and wavelet (wavelet-LHL, wavelet-LHH, wavelet-HLL, wavelet-LLH, wavelet-HLH, wavelet-HHH, wavelet-HHL, and wavelet-LLL).

Inter- and intra- class correlation coefficients (ICCs) were used to evaluate the inter-observer reliability and intra-observer reproducibility of feature extraction. We randomly chose 30 cases of CT images (10 FNHs and 20 HCCs), and ROI segmentation was performed by Reader 1 and Reader 2. Reader 1 then repeated the same procedure 1 week later to evaluate the agreement of feature extraction. An ICC greater than 0.75 suggests good agreement of the feature extraction. The remaining image segmentation was conducted by Reader 1.

### Construction of the radiomics signature

The radiomics features, which met the criteria of having inter- and intraobserver ICCs greater than 0.75 and being significantly different between the two groups evaluated by one-way analysis of variance (ANOVA), were entered into the least absolute shrinkage and selection operator (LASSO) regression model to select the most valuable features in the training set. The selected features were then combined into a radiomics signature. A radiomics score (Rad-score) was calculated for each patient through a linear combination of selected features weighted by their respective LASSO coefficients.

### Development of a radiomics nomogram and assessment of the performance of different models

A radiomics nomogram was developed by incorporating the significant variables of the clinical factors as well as the Rad-score. The diagnostic performance of the clinical factors model, the radiomics signature and the radiomics nomogram for differentiating FNH from HCC was assessed by using the area under the receiver operator characteristic (ROC) curve (AUC) in both the training and validation sets. A radiomics nomogram-defined score (Nomo-score) for each patient was calculated in the training and validation sets. To estimate the clinical utility of the nomogram, decision curve analysis (DCA) was performed by calculating the net benefits for a range of threshold probabilities in the training set.

### Statistics

Statistical analysis was performed using SPSS (Version 25.0, IBM) and R statistical software (Version 3.3.3, https://www.r-project.org). Univariate analysis was used to compare the differences of the clinical factors between the two groups by using the chi-square test or Fisher exact test for categoric variables, and Mann-Whitney U test for continuous variables, where appropriate. One-way ANOVA was used to compare the value of radiomics features for differentiation of FNH and HCC. The “glmnet” package was used to perform the LASSO regression model analysis. The ROC curves were plotted using the “pROC” package. Nomogram construction was performed using the “rms” package. Differences in the AUC values between these models were analyzed using the Delong test. DCA was performed using the “dca. R.” package. *P* < 0.05 was considered statistically significant.

## Results

### Clinical factors of the patients and the construction of the clinical factors model

The clinical factors of the patients in the training and validation sets are shown in Table 1. There was significant difference in age, gender, HBV infection, AFP level, central scar, degeneration, capsule-like rim and enhancement pattern between the two groups (*P* < 0.05), whereas diameter, shape, fat deposition, calcification, and dysmorphic vessels were not significantly different between the two groups (*P* > 0.05) in the training set.

**Table 1:** Clinical factors of the training and validation sets

Clinical factors	Training set (*n* = 119)			Validation set (*n* = 37)		
	FNH (*n* = 42)	HCC (*n* = 77)	P	FNH (*n* = 13)	HCC (*n* = 24)	P
Gender (Male/Female)	22/20	63/14	0.001	10/3	22/2	0.321
Age (Median [range]), year	32(2–62)	58(33–78)	0.000	28(10–69)	59(36–78)	0.000
HbsAg (Positive/Negative)	1/41	60/17	0.000	1/12	19/5	0.000
HcsAg (Positive/Negative)	0/42	0/77	–	0/13	0/24	–
AFP (> 400 ng/mL/≤ 400 ng/mL)	0/42	22/55	0.000	1/12	5/19	0.394
Diameter (Median [range]), milimetre	42(12–87)	44 (11–164)	0.982	39(16–134)	39(21–204)	0.888
Shape (Round/Not round)	30/12	55/22	1.000	9/4	20/4	0.413
Central scar (Present/Absent)	19/23	8/69	0.000	2/11	2/22	0.602
Degeneration (Present/Absent)	5/37	48/29	0.000	3/10	14/10	0.082
Fat deposition (Present/Absent)	0/42	1/76	1.000	0/13	0/24	–
Calcification (Present/Absent)	0/42	2/75	0.539	0/13	0/24	–
Capsule-like rim (Present/Absent)	1/41	21/56	0.000	0/13	5/19	0.140
Dysmorphic vessels (Present/Absent)	23/19	30/47	0.123	5/8	10/14	1.000
Enhancement pattern			0.000			0.000
Early enhancement + washout	4	69	–	4	22	–
Early enhancement + no washout	37	2	–	9	0	–
Other patterns	1	6	–	0	2	–

Note: *FNH* (Focal nodular hyperplasia); *HCC* (Hepatocellular carcinoma); *HbsAg* (Hepatitis B surface antigen); *HcsAg* (Hepatitis C surface antigen); *AFP* (Alpha fetoprotein)

The multiple logistic regression analysis showed that only age (*P* < 0.001), HBV infection (*P* = 0.001), and enhancement pattern (*P* = 0.019) remained as independent predictors in the clinical factors model. Tumours with older age (Odds ratio [OR], 0.818; 95% CI, 0.736–0.909), HBV infection (OR, 68.580; 95% CI, 5.154–912.560) or early enhancement with a washout pattern (OR, 4.905; 95% CI, 1.305–18.431) were likely to be HCCs.

### Feature extraction, selection and radiomics signature construction

**Fig. 1:**
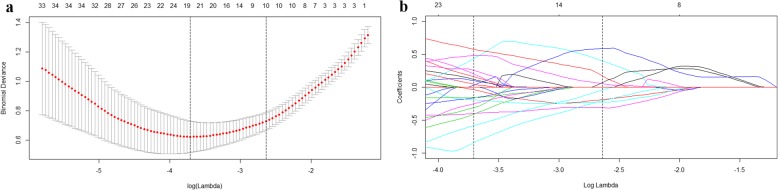
Radiomics feature selection using logistic regression with the least absolute shrinkage and selection operator (LASSO) regularization. (**a**) Ten-fold cross-validation via minimum criteria was used to select the tuning parameter (λ) in LASSO model. The optimal values of the LASSO tuning parameter (λ) are indicated by the dotted vertical lines. An optimal λ value of 0.0714, with log(λ) = − 2.6394 was chosen (**b**) LASSO coefficient profiles of the 764 raidomics features. A coefficient profile plot was generated versus the selected log (λ) value using ten-fold cross validation, the vertical line was plotted with 10 selected radiomics features

Of the 4227 radiomics features extracted from AP, PVP and DP CT images, 3441 were shown to have a good inter- and intra-observer agreement, with ICCs from 0.750 to 1.000. 764 radiomics features having significant differences between FNH and HCC (*P* = 0.001–0.050) were entered into the LASSO logistic regression model to select the most valuable features (Fig.1). Finally, the radiomics signature was built by using 10 features. The Rad-score was calculated using the following formula:

Rad-score = 0.0522 × GrayLevelNonUniformityNormalized.glrlm.logarithm. AP-0.3125 × Imc2.glcm.wavelet_LLL.DP-0.1885 × JointAverage.glcm.wavelet_LHH.DP-0.2271 × Mean.firstorder.wavelet_HLL.DP + 0.3989 × Median.firstorder.wavelet_LLL.DP-0.2055 × Skewness.firstorder.wavelet_LLL.AP + 0.0700 × Uniformity.firstorder.logarithm. PVP + 0.1703 × Uniformity.firstorder.squareroot. PVP + 0.5864 × X10Percentile.firstorder.exponential. AP-0.0404 × X10Percentile.firstorder.wavelet_HLL.DP. (AP [arterial phase], PVP [portal venous phase], and DP [delayed phase]).

The Rad-score showed statistically significant differences between FNH and HCC (median Rad-score of FNH: 1.580, range: [− 0.543, 4.326]; median Rad-score of HCC: − 0.834, range: [− 3.073, 1.022]; *P* < 0.001 in the training set and median Rad-score of FNH: 0.837, range: [− 1.460, 3.987]; median Rad-score of HCC: − 0.763, range: [− 2.643, 1.498]; P < 0.001 in the validation set).

### The radiomics nomogram building and assessment of the performance of different models

**Fig. 2:**
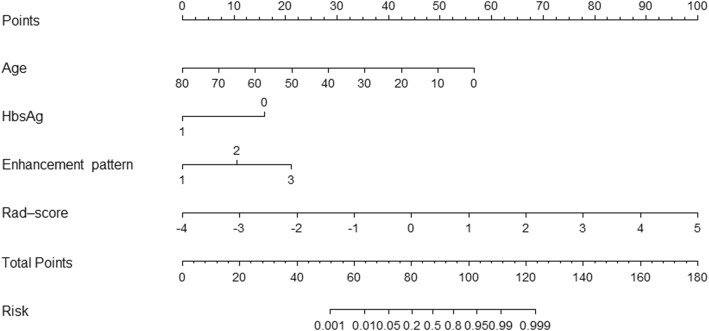
The radiomics nomogram, combining age, HBV infection, enhancement pattern, and Radiomics score (Rad-score), developed in the training set. Enhancement pattern 1, 2, and 3 represented early enhancement with a washout pattern, early enhancement with no washout pattern, and other enhancement patterns, respectively

**Fig. 3:**
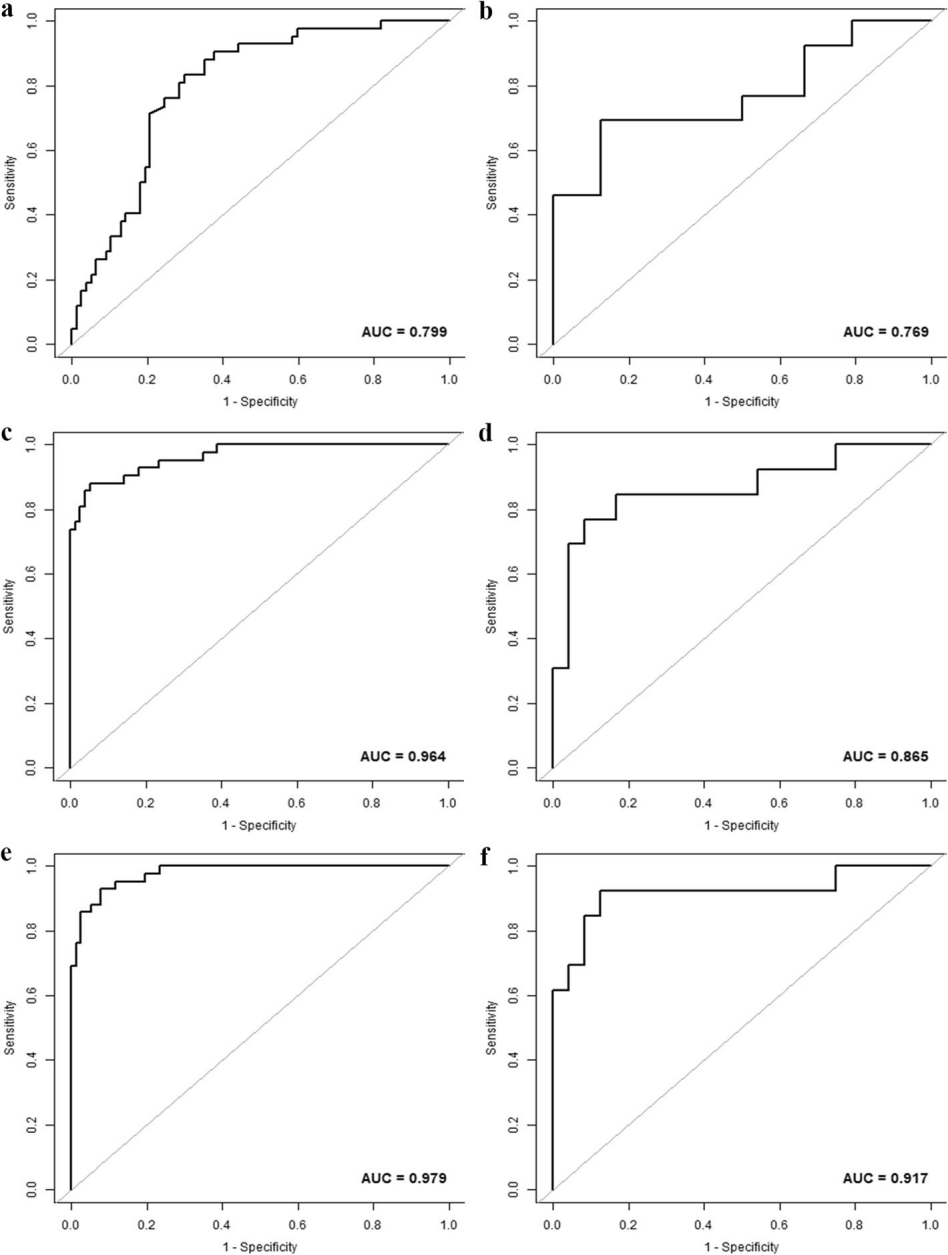
The ROC curves of the clinical factors model (**a**, **b**), the radiomics signature (**c**, **d**) and the radiomics nomogram (**e**, **f**) in the training and validation sets, respectively

**Fig. 4:**
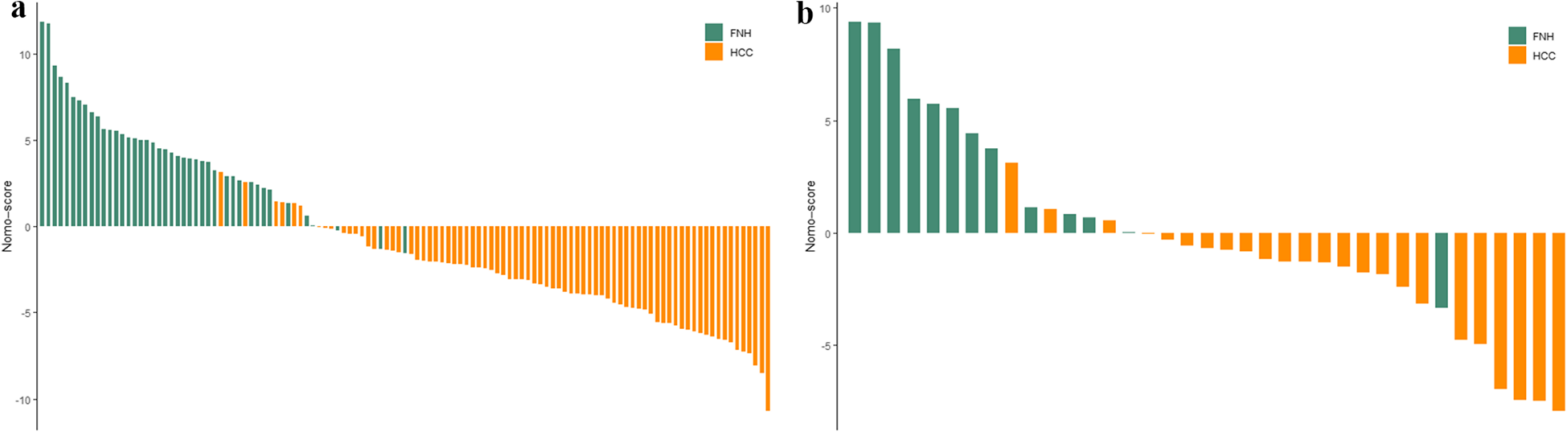
The radiomics nomogram-defined scores for each patient in the training and validation sets. Orange bars represent the scores for HCC patients, while green bars represent the scores for FNH patients

**Fig. 5:**
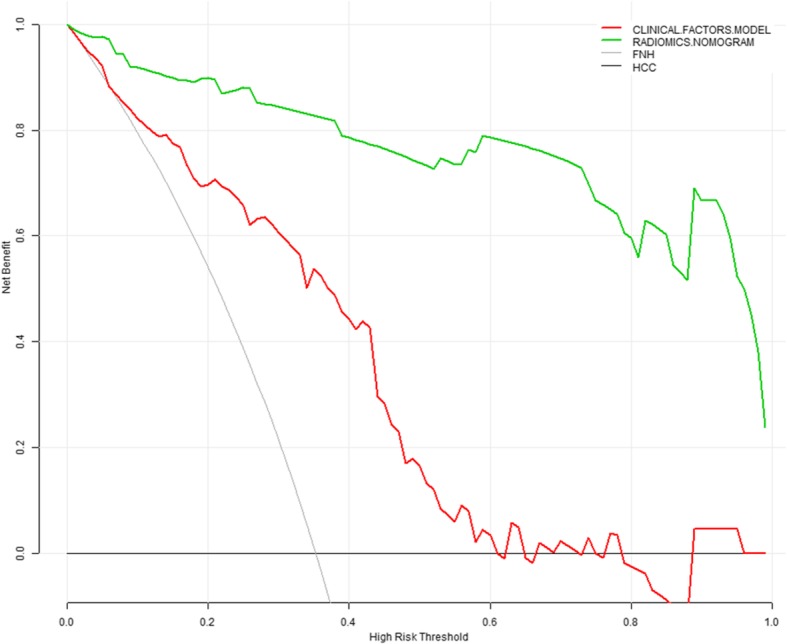
Decision curve analysis for the radiomics nomogram and the clinical factors model. The y-axis indicates the net benefit; x-axis indicates threshold probability. The horizontal black line represents the assumption of all HCC patients, while the grey line represents the assumption of all FNH patients. Based on the threshold probabilities obtained, our findings indicated that the radiomics nomogram (green line) provided a greater net benefit than the clinical factors model (red line)

The age, HBV infection, enhancement pattern, and Rad-score were incorporated into the radiomics nomogram building (Fig. 2). The diagnostic performance for the clinical factors model, the radiomics signature and the radiomics nomogram is summarized in Table 2. ROC curves of the three models are shown in Fig. 3. In the training set, the AUCs of the radiomics nomogram and the radiomics signature were significantly higher than that of the clinical factors model (both *P* < 0.001); however, no significant difference in AUC was found between the radiomics nomogram and the radiomics signature (*P* = 0.253). In the validation set, there were no significant differences in AUC among these three models (the clinical factors model vs. the radiomics signature, *P* = 0.376; the clinical factors model vs. the radiomics nomogram, *P* = 0.055; the radiomics signature vs. the radiomics nomogram, *P* = 0.345). The Nomo-scores for each patient in the training and validation sets are shown in Fig. 4. The DCA (Fig. 5) showed the radiomics nomogram had a higher overall net benefit in differentiating FNH from HCC than the clinical factors model across the majority of the range of reasonable threshold probabilities.

**Table 2:** Diagnostic Performance of the clinical factors model, the radiomics signature and the radiomics nomogram for detection of focal nodular hyperplasia

Model	Training set (n = 119)					Validation set (n = 37)				
	Cutoff	AUC (95% CI)	Sensitivity^*^	Specificity^*^	Accuracy^*^	Cutoff	AUC (95% CI)	Sensitivity^*^	Specificity^*^	Accuracy^*^
Clinical factors model	−1.513	0.799(0.719,0.879)	83.33(35/42)	70.13(54/77)	74.79(89/119)	−0.434	0.769(0.590,0.949)	69.23(9/13)	87.50(21/24)	81.08(30/37)
Radiomics signature	0.543	0.964(0.934,0.995)	88.10(37/42)	94.81(73/77)	92.44(110/119)	−0.306	0.865(0.725,1.000)	76.92(10/13)	91.67(22/24)	86.49(32/37)
Radiomics nomogram	−1.405	0.979(0.959,0.998)	92.86(39/42)	92.21(71/77)	92.44(110/119)	−2.795	0.917(0.800,1.000)	92.31(12/13)	87.50(21/24)	89.19(33/37)

Note: CI (Confidence interval); ^*^ Numbers in parentheses were used to calculate percentages

## Discussion

The present study shows that the enhanced CT-based radiomics nomogram, which incorporates the radiomics signature and clinical factors, has favorable predictive value for differentiating HCC from FNH in the non-cirrhotic liver with the AUC of 0.979 and 0.917, respectively in the training set and validation set.

Differentiating HCC from FNH is important to select appropriate treatment and avoid unnecessary interventions. Sufficient clinical and imaging information facilitates the correct distinction of the two lesions. FNH occurs more frequently in young women (male:female ratio = 1:8) [4, 7, 45]. HCC is often associated with hepatitis virus infection and a higher level of AFP. Five clinical data including age, gender, hepatitis B and C virus infection and serum AFP level were analyzed in this study; and we found that FNH patients had a significantly younger age and female predominance compared with the HCC counterpart, while in the HCC group, there were more hepatitis B virus infectors associated with a higher AFP level. Age and HBV infection were proven as independent predictors by using the multiple logistic regression analysis, which was consistent with previous studies.

Contrast-enhanced CT (CECT) is the first-line imaging modality for the characterization of liver lesions. However, distinguishing an FNH from an HCC on CECT remains a challenge, especially when they lack typical imaging characteristics such as a central scar, suggestive of FNH (reported in about 65% of FNHs larger than 3 cm [6]) or liver cirrhosis, suggestive of HCC. HCC shares overlapping imaging features with FNH in the non-cirrhotic liver. The classic radiological hallmark of HCC is a hyperenhancement on AP and PVP or DP washout. FNH may also present as a hypervascular lesion with intense enhancement and washout on PVP and DP. Therefore, CECT has a limited diagnostic value in the non-cirrhotic liver for the distinction of HCC and atypical FNH.

Various strategies have been proposed to differentiate benign from malignant liver tumours with conventional CT and MR imaging characteristics. Yu et al. [9] enrolled 42 HCCs and 16 FNHs to identify the value of CT spectral imaging in differentiating HCC from FNH during the AP and PVP, and found that the lesion-normal parenchyma iodine concentration ratio in AP had the highest sensitivity (100%) and specificity (100%) in differentiating HCC from FNH. Boas et al. [10] developed and validated a simplified triphasic CT-based model of tumor blood supply that combined hepatic artery and portal vein blood supply coefficients to distinguish benign (*n* = 32) and malignant (*n* = 46) liver lesions. In addition to traditional relative enhancement criteria (such as washout), hepatic artery and portal vein blood supply coefficients could be used to classify hypervascular liver lesions, achieving high specificity (97%) and high sensitivity (76%) for malignancy. Fischer et al. [3] included 55 HCCs, 28 FNHs, and 24 HCAs to identify the key MRI features that can potentially be used to differentiate between HCC and benign hepatocellular tumors in the non-cirrhotic liver. Multivariate analysis revealed T1-hypointensity, T2-hypo- or hyperintensity, lack of central tumor-enhancement, presence of satellite-lesions, and lack of liver-specific contrast media uptake were independent MRI features indicating HCC. Kitao et al. [1] tried to identify points useful in the imaging differentiation of HCC, showing hyperintensity on the HBP of gadoxetic acid-enhanced MRI and FNH and FNH-like nodules. The CT and MRI features of 51 HCCs, 10 FNHs, and 16 FNH-like nodules were analyzed. Multivariate logistic regression analysis showed that arterial phase enhancement and washout pattern at dynamic CT and decrease of ADC ratio would be important findings for the diagnosis of hyperintense HCC differentiated from FNH and FNH-like nodule. In the present study, a clinical factors model was developed combining clinical data with subjective CT features by using multivariate logistic regression analysis, and age, HBV infection, and enhancement pattern were found as significant predictors for differential diagnosis. By using this clinical factors model, high AUC (0.799 in training set; 0.769 in the validation set) for differential diagnosis of HCC from FNH were achieved.

Radiomics enables the noninvasive profiling of tumor heterogeneity by extracting high throughput of quantitative descriptors from routinely acquired CT and MRI studies. Previous investigations have shown that CT/MRI-based radiomics can be used for differentiating several hypervascular liver tumours. Raman et al. [41] demonstrated that CT texture analysis could be used to distinguish different hypervascular liver lesions using a random-forest model. Seventeen FNHs, 19 HCAs, 25 HCCs, and 19 cases of normal liver parenchyma were analyzed, and the texture model successfully distinguished the three lesion types and normal liver with predicted classification performance accuracy for 91.2% for HCA, 94.4% for FNH, and 98.6% for HCC. Wu et al. [37] developed and validated an MRI-based radiomics signature to distinguish HCC and HH using four feature classifiers, and found that the logistic regression classifier showed better predictive ability, achieving an AUC of 0.89 for differentiating HCC from HH. Stocker et al. [38] enrolled 55 cases of HCC and 45 cases of benign hepatocellular tumors (including 24 HCAs and 29 FNHs) in the non-cirrhotic liver to assess the accuracy of MRI texture features in differentiating benign from malignant liver tumours. One gray-level histogram (skewness) and four run-length matrix features extracted from AP images were regarded as the significant texture predictors aiding distinguishing HCC from benign hepatocellular tumors in the non-cirrhotic liver with an accuracy of 84.5% and an AUC of 0.92. Cannella et al. [43] investigated the texture and subjective MRI features of 32 FHNs and 51 HCAs and found that MRI TA parameters combined with hypointensity on HBP imaging yielded an AUC of 0.979 and an accuracy of 96.4% for the diagnosis of HCA. A similar CT-based texture model was built by Cannella et al. [42] for the distinction of HCA and FNH. The mean, mpp, and entropy of medium-level and coarse-level filtered images on AP were found as independent predictors for the diagnosis of HCA and the model based on all these parameters resulted in the largest AUC of 0.824. In this study, a radiomics nomogram was constructed by combining age, HBV infection, enhancement pattern, and Rad-score. The multiple logistic regression analysis showed that the Rad-score made a major contribution to differential diagnosis. In these independent clinical predictors, age provided much more weightage than enhancement pattern. The result was consistent with previous studies that HCC occurred more frequently in older patients compared with FNH [4, 7, 45]. Although their enhancement patterns are significantly different, the two entities share overlapping enhancement features [6]. The enhancement pattern has a limited impact on the distinction between HCC and FNH in the non-cirrhotic liver.

Compared to the above radiomics investigations on discrimination of different hepatic tumours, our study had several improvements. First, we chose to focus on the distinction of FNH and HCC in the non-cirrhotic liver, because these tumours are the most difficult to differentiate in routine clinical practice and are often the cause of diagnostic errors. Second, previous studies were mainly based on texture analysis associated with only dozens of texture features. Nowadays, radiomics with much more statistic features are available to provide a more comprehensive description of the tumour. In this study, a total of 4227 radiomics features were extracted and analyzed from the triphasic CT images, and finally, 10 features were selected as the significant predictors to construct the radiomics signature. All the selected features were high-order filter and wavelet features that could not be acquired by using conventional texture analysis. Furthermore, both AP, PVP, and DP CT images were used for feature selection, and 5/10 of selected features were obtained from DP images, indicating a trend toward better lesion classification with DP images for FNH and HCC. In addition, FNH is not associated with any malignant potential, and most lesions are managed conservatively. The FNH confirmed with surgical pathology only accounts for a small portion of the whole cohort. The cases of FNH enrolled in the present study were relatively more than those in previous studies.

We acknowledge the following limitations in our study. First, because of its retrospective character, potential selection bias may hamper the reproducibility and comparability of the results. Thus, the clinical usefulness of this nomogram still needs improvement and independent validation in further studies. Second, this study was a single-center experience limited to our institute, multi-center studies for further validation of its reproducibility with a larger sample are required. Third, the two-dimensional largest tumorous ROIs were delineated for the extraction of radiomics features. Whole tumour analysis appears more indicative of tumour heterogeneity than the largest cross-sectional area [46]. In addition, manual ROI segmentation is time-consuming and complicated, especially for the tumour without a well-defined boundary, the automatic segmentation technique with favorable reliability and reproducibility is needed. Fourth, it is reported that slice thickness can affect the diagnostic performance of radiomics signature, and the thin slice may be more informative [47]. A slice thickness of 5 mm was used in this study, which is usually thick for CT radiomics analysis. The difference of the performance in radiomics analysis between the thin and thick slice thickness images will be assessed in our future study.

## Conclusions

In conclusion, the CT-based radiomics nomogram developed and validated for preoperative differentiation of FNH from HCC in the non-cirrhotic liver can potentially supplement conventional imaging modalities. However, the clinical use of this tool remains to be tested.

## Data Availability

The datasets generated and analysed during the current study are not publicly available due to patient privacy concerns but are available from the corresponding author on reasonable request approved by the institutional review boards of the Affiliated Hospital of Qingdao University.

## Abbreviations

AFP: Alpha fetoprotein
ANOVA: Analysis of variance
AP: Arterial phase
AUC: Area under the curve
CECT: Contrast-enhanced CT
CI: Confidence interval
DP: Delayed phase
FNH: Focal nodular hyperplasia
GLCM: Gray level co-occurence matrix
GLRLM: Gray level run length matrix
GLSZM: Gray level size zone matrix
HBP: Hepatobiliary phase
HCA: Hepatocellular adenoma
HCC: Hepatocellular carcinoma
HH: Hepatic haemangioma
ICC: Inter−/intra- class correlation coefficient
ICC: Intrahepatic cholangiocarcinoma
LASSO: Least absolute shrinkage and selection operator
Nomo-score: Nomogram-defined score
OATP: Organic anion-transporting polypeptide
OR: Odds ratio
PACS: Picture archiving and communication system
PVP: Portal venous phase
Rad-score: Radiomics score
ROC: Receiver operator characteristic
ROI: Region of interest

## References

[1] Kitao A, Matsui O, Yoneda N, Kita R, Kozaka K, Kobayashi S (2018). Differentiation between hepatocellular carcinoma showing hyperintensity on the hepatobiliary phase of gadoxetic acid-enhanced MRI and focal nodular hyperplasia by CT and MRI. AJR Am J Roentgenol.

[2] Kamaya A, Maturen KE, Tye GA, Liu YI, Parti NN, Desser TS (2009). Hypervascular liver lesions. Semin Ultrasound CT MR.

[3] Fischer MA, Raptis DA, Donati OF, Hunziker R, Schade E, Sotiropoulos GC (2015). MR imaging features for improved diagnosis of hepatocellular carcinoma in the non-cirrhotic liver: multi-center evaluation. Eur J Radiol.

[4] Virgilio E, Cavallini M (2018). Managing focal nodular hyperplasia of the liver: surgery or minimally-invasive approaches? A review of the preferable treatment options. Anticancer Res.

[5] Kim JW, Lee CH, Kim SB, Park BN, Park YS, Lee J (2017). Washout appearance in Gd-EOB-DTPA-enhanced MR imaging: a differentiating feature between hepatocellular carcinoma with paradoxical uptake on the hepatobiliary phase and focal nodular hyperplasia-like nodules. J Magn Reson Imaging.

[6] Dioguardi Burgio M, Ronot M, Salvaggio G, Vilgrain V, Brancatelli G (2016). Imaging of hepatic focal nodular hyperplasia: pictorial review and diagnostic strategy. Semin Ultrasound CT MR.

[7] Khanna M, Ramanathan S, Fasih N, Schieda N, Virmani V, McInnes MD (2015). Current updates on the molecular genetics and magnetic resonance imaging of focal nodular hyperplasia and hepatocellular adenoma. Insights Imaging.

[8] Grazioli L, Bondioni MP, Faccioli N, Gambarini S, Tinti R, Schneider G (2010). Solid focal liver lesions: dynamic and late enhancement patterns with the dual phase contrast agent gadobenate dimeglumine. J Gastrointest Cancer.

[9] Yu Y, Lin X, Chen K, Chai W, Hu S, Tang R (2013). Hepatocellular carcinoma and focal nodular hyperplasia of the liver: differentiation with CT spectral imaging. Eur Radiol.

[10] Boas FE, Kamaya A, Do B, Desser TS, Beaulieu CF, Vasanawala SS (2015). Classification of hypervascular liver lesions based on hepatic artery and portal vein blood supply coefficients calculated from triphasic CT scans. J Digit Imaging.

[11] Zheng SG, Xu HX, Liu LN, Wang Y, Zhang YF, Guo LH (2013). Parametric imaging with contrast-enhanced ultrasound: usefulness for characterization of dynamic effects of microvascularization for hepatocellular carcinoma and focal nodular hyperplasia. Clin Hemorheol Microcirc.

[12] Pei XQ, Liu LZ, Xiong YH, Zou RH, Chen MS, Li AH (2013). Quantitative analysis of contrast-enhanced ultrasonography: differentiating focal nodular hyperplasia from hepatocellular carcinoma. Br J Radiol.

[13] Zarghampour M, Fouladi DF, Pandey A, Ghasabeh MA, Pandey P, Varzaneh FN (2018). Utility of volumetric contrast-enhanced and diffusion-weighted MRI in differentiating between common primary hypervascular liver tumors. J Magn Reson Imaging.

[14] Onur MR, Cicekci M, Kayali A, Poyraz AK, Kocakoc E (2012). The role of ADC measurement in differential diagnosis of focal hepatic lesions. Eur J Radiol.

[15] Haimerl M, Wächtler M, Platzek I, Müller-Wille R, Niessen C, Hoffstetter P (2013). Added value of Gd-EOB-DTPA-enhanced hepatobiliary phase MR imaging in evaluation of focal solid hepatic lesions. BMC Med Imaging.

[16] Saini Aman, Breen Ilana, Pershad Yash, Naidu Sailendra, Knuttinen M., Alzubaidi Sadeer, Sheth Rahul, Albadawi Hassan, Kuo Malia, Oklu Rahmi (2018). Radiogenomics and Radiomics in Liver Cancers. Diagnostics.

[17] Gillies RJ, Kinahan PE, Hricak H (2016). Radiomics: images are more than pictures, they are data. Radiology.

[18] Shan QY, Hu HT, Feng ST, Peng ZP, Chen SL, Zhou Q (2019). CT-based peritumoral radiomics signatures to predict early recurrence in hepatocellular carcinoma after curative tumor resection or ablation. Cancer Imaging.

[19] Oh J, Lee JM, Park J, Joo I, Yoon JH, Lee DH (2019). Hepatocellular carcinoma: texture analysis of preoperative computed tomography images can provide markers of tumor grade and disease-free survival. Korean J Radiol.

[20] Kim S, Shin J, Kim DY, Choi GH, Kim MJ, Choi JY. Radiomics on gadoxetic acid-enhanced magnetic resonance imaging for prediction of postoperative early and late recurrence of single hepatocellular carcinoma. Clin Cancer Res. 2019. 10.1158/1078-0432. 10.1158/1078-0432.CCR-18-2861 30808773

[21] Cai W, He B, Hu M, Zhang W, Xiao D, Yu H (2019). A radiomics-based nomogram for the preoperative prediction of posthepatectomy liver failure in patients with hepatocellular carcinoma. Surg Oncol.

[22] Zheng BH, Liu LZ, Zhang ZZ, Shi JY, Dong LQ, Tian LY (2018). Radiomics score: a potential prognostic imaging feature for postoperative survival of solitary HCC patients. BMC Cancer.

[23] Zhang Jing, Liu Xinjie, Zhang Haiping, He Xiaojing, Liu Yangyang, Zhou Jun, Guo Dajing (2019). Texture Analysis Based on Preoperative Magnetic Resonance Imaging (MRI) and Conventional MRI Features for Predicting the Early Recurrence of Single Hepatocellular Carcinoma after Hepatectomy. Academic Radiology.

[24] Yao Z, Dong Y, Wu G, Zhang Q, Yang D, Yu JH (2018). Preoperative diagnosis and prediction of hepatocellular carcinoma: Radiomics analysis based on multi-modal ultrasound images. BMC Cancer.

[25] Wu M, Tan H, Gao F, Hai J, Ning P, Chen J (2019). Predicting the grade of hepatocellular carcinoma based on non-contrast-enhanced MRI radiomics signature. Eur Radiol.

[26] Mulé S, Thiefin G, Costentin C, Durot C, Rahmouni A, Luciani A (2018). Advanced hepatocellular carcinoma: pretreatment contrast-enhanced CT texture parameters as predictive biomarkers of survival in patients treated with sorafenib. Radiology.

[27] Kim J, Choi SJ, Lee SH, Lee HY, Park H (2018). Predicting survival using pretreatment CT for patients with hepatocellular carcinoma treated with transarterial chemoembolization: comparison of models using radiomics. AJR Am J Roentgenol.

[28] Jeong WK, Jamshidi N, Felker ER, Raman SS, Lu DS (2019). Radiomics and radiogenomics of primary liver cancers. Clin Mol Hepatol.

[29] Hui TCH, Chuah TK, Low HM, Tan CH (2018). Predicting early recurrence of hepatocellular carcinoma with texture analysis of preoperative MRI: a radiomics study. Clin Radiol.

[30] Brenet Defour L, Mule S, Tenenhaus A, Piardi T, Sommacale D, Hoeffel C (2019). Hepatocellular carcinoma: CT texture analysis as a predictor of survival after surgical resection. Eur Radiol.

[31] Akai H, Yasaka K, Kunimatsu A, Nojima M, Kokudo T, Kokudo N (2018). Predicting prognosis of resected hepatocellular carcinoma by radiomics analysis with random survival forest. Diagn Interv Imaging.

[32] Zhou Y, He L, Huang Y, Chen S, Wu P, Ye W (2017). CT-based radiomics signature: a potential biomarker for preoperative prediction of early recurrence in hepatocellular carcinoma. Abdom Radiol (NY).

[33] Chen S, Zhu Y, Liu Z, Liang C (2017). Texture analysis of baseline multiphasic hepatic computed tomography images for the prognosis of single hepatocellular carcinoma after hepatectomy: a retrospective pilot study. Eur J Radiol.

[34] Ma Xiaohong, Wei Jingwei, Gu Dongsheng, Zhu Yongjian, Feng Bing, Liang Meng, Wang Shuang, Zhao Xinming, Tian Jie (2019). Preoperative radiomics nomogram for microvascular invasion prediction in hepatocellular carcinoma using contrast-enhanced CT. European Radiology.

[35] Hu HT, Wang Z, Huang XW, Chen SL, Zheng X, Ruan SM (2019). Ultrasound-based radiomics score: a potential biomarker for the prediction of microvascular invasion in hepatocellular carcinoma. Eur Radiol.

[36] Xu X, Zhang HL, Liu QP, Sun SW, Zhang J, Zhu FP (2019). Radiomic analysis of contrast-enhanced CT predicts microvascular invasion and outcome in hepatocellular carcinoma. J Hepatol.

[37] Wu J, Liu A, Cui J, Chen A, Song Q, Xie L (2019). Radiomics-based classification of hepatocellular carcinoma and hepatic haemangioma on precontrast magnetic resonance images. BMC Med Imaging.

[38] Stocker D, Marquez HP, Wagner MW, Raptis DA, Clavien PA, Boss A (2018). MRI texture analysis for differentiation of malignant and benign hepatocellular tumors in the non-cirrhotic liver. Heliyon.

[39] Li Z, Mao Y, Huang W, Li H, Zhu J, Li W (2017). Texture-based classification of different single liver lesion based on SPAIR T2W MRI images. BMC Med Imaging.

[40] Suo ST, Zhuang ZG, Cao MQ, Qian LJ, Wang X, Gao RL (2016). Differentiation of pyogenic hepatic abscesses from malignant mimickers using multislice-based texture acquired from contrast-enhanced computed tomography. Hepatobiliary Pancreat Dis Int.

[41] Raman SP, Schroeder JL, Huang P, Chen Y, Coquia SF, Kawamoto S (2015). Preliminary data using computed tomography texture analysis for the classification of hypervascular liver lesions: generation of a predictive model on the basis of quantitative spatial frequency measurements-a work in progress. J Comput Assist Tomogr.

[42] Cannella R, Borhani AA, Minervini MI, Tsung A, Furlan A (2019). Evaluation of texture analysis for the differential diagnosis of focal nodular hyperplasia from hepatocellular adenoma on contrast-enhanced CT images. Abdom Radiol (NY).

[43] Cannella R, Rangaswamy B, Minervini MI, Borhani AA, Tsung A, Furlan A (2019). Value of texture analysis on gadoxetic acid-enhanced MRI for differentiating hepatocellular adenoma from focal nodular hyperplasia. AJR Am J Roentgenol.

[44] Lewis S, Peti S, Hectors SJ, King M, Rosen A, Kamath A (2019). Volumetric quantitative histogram analysis using diffusion-weighted magnetic resonance imaging to differentiate HCC from other primary liver cancers. Abdom Radiol (NY).

[45] Yoneda N, Matsui O, Kitao A, Kozaka K, Kobayashi S, Sasaki M (2016). Benign hepatocellular nodules: hepatobiliary phase of gadoxetic acid-enhanced MR imaging based on molecular background. Radiographics.

[46] Ng F, Kozarski R, Ganeshan B, Goh V (2013). Assessment of tumor heterogeneity by CT texture analysis: can the largest cross-sectional area be used as an alternative to whole tumor analysis?. Eur J Radiol.

[47] He L, Huang Y, Ma Z, Liang C, Liang C, Liu Z (2016). Effects of contrast-enhancement, reconstruction slice thickness and convolution kernel on the diagnostic performance of radiomics signature in solitary pulmonary nodule. Sci Rep.

